# Anthropometric measurements in Ponseti treated clubfeet

**DOI:** 10.1051/sicotj/2018010

**Published:** 2018-05-25

**Authors:** Anil Agarwal, Anuj Rastogi

**Affiliations:** 1 Pediatric Orthopedic Surgeon, Department of Pediatric Orthopaedics, Chacha Nehru Bal Chikitsalaya, Geeta Colony, Delhi 31 India; 2 Fellow, Indian Orthopaedic Association, Johari Medical Research Foundation (IOA- JMRF 2016), Department of Pediatric Orthopaedics, Chacha Nehru Bal Chikitsalaya, Geeta Colony, Delhi 31 India

**Keywords:** Foot size, Length width, Dimensions, Clubfoot, CTEV

## Abstract

*Introduction*: We measured the foot dimensions in the Ponseti treated idiopathic clubfeet to compare differences in foot sizes, if any.

*Patient and Methods*: The foot length and width in unaffected, unilateral and bilateral clubfeet were measured and analysed statistically.

*Results*: Average follow up was 22.2 months. Bilateral feet were similar in size. The unilateral affected feet matched in size with contralateral unaffected feet. The size difference between bilateral and unilateral affected feet was not significant. The bilateral feet were significantly smaller than age matched unaffected feet [in length 0.8 cm (6.1%); *p* = 0.03 and in width 0.2 cm, (3.7%); *p* = 0.03]. The unilateral foot was comparable with contralateral unaffected foot both during and post bracing.

*Conclusions*: Post Ponseti treatment, inter bilateral, unilateral affected versus unaffected, bilateral versus unilateral affected feet matched in size. The overall clubfeet size especially those with bilateral disease were significantly shorter than unaffected side. The Ponseti managed unilateral foot size was comparable with unaffected foot during the bracing duration and size comparability was maintained even after bracing protocol of 3 years was over.

## Introduction

Ponseti method for treatment of clubfoot has revolutionized the clubfoot management. Long term studies on Ponseti method have effectively demonstrated the excellent to good outcome of the affected feet using clinical, radiographic, functional, gait, pedobarographic and electrogoniometric analyses [1,2]. Numerous testimonials certify that patients with clubfeet are venturing into all jobs and challenging sports [3]. The newer age patients and their parents are also concerned regarding foot aesthetics and footwear size in long term besides adequate functional results. The clubfoot information on the several internet sites still describe the affected foot to be on average 1–1½ size smaller [4,5]. These beliefs probably originated when extensive surgeries were the predominant modality for clubfoot treatment. Except for numerous scientific papers on calf size in clubfoot, the data on foot size remains scanty [6–9].

We measured the foot dimensions in the Ponseti treated idiopathic clubfeet to compare differences in foot sizes, if any. We further investigated that whether there were any variations in foot size in the prescribed Steenbeek foot abduction brace (SFAB) use of 3 years and the post bracing period.

## Material and methods

This cross sectional study was conducted on follow up patients with corrected idiopathic clubfeet using Ponseti method visiting our CURE Clubfoot Clinic (1st–30th July 2016). Our institutional protocol defines the total bracing duration of 3 years post tenotomy post correction [10]. The study included patients using SFAB for minimum 3 months or beyond. Thus, the study also included patients who have already completed the prescribed bracing protocol of 3 years but were still available for follow up.

## Measurements

The measurements were based on the anthropometric method described by Kesemenli et al. [8]. In each child, both feet were measured after marking the outline of child's foot placed flat on a white paper sheet secured on a board and measured with a millimetre scale measure. All the measurements were taken by a single orthopaedic surgeon during the morning outpatient clinics using the same device. A full and free consent was obtained from patients' relatives for the study to be published, to which they did not have any objection.

**Figure 1:**
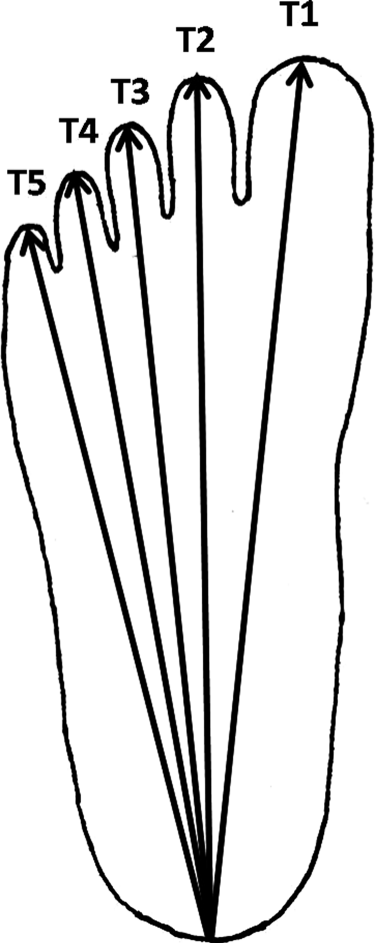
Measurement of foot size: the distal tip of toe to heel distance was marked on the paper and measured. The mean of the measurements T1–T5 was taken as foot length. Distance between the outer margins of the first and fifth tarsometatarsal joints was measured as foot width.

– Toe lengths (Figure 1):
  - T1: Distance between the proximal of the calcaneal tubercle (Calcaneus) and tip of hallux
  - T2: Distance between the calcaneus and tip of second toe
  - T3: Distance between the calcaneus and tip of third toe
  - T4: Distance between the calcaneus and tip of fourth toe
  - T5: Distance between the calcaneus and tip of fifth toe
– Foot width (FW): Distance between the outer margins of the first and fifth tarsometatarsal joints.

## Calculations and statistical methods

There were a total of 100 patients with 51 unilateral (26 left + 25 right) and 49 bilateral feet. The patients in all three groups- bilateral, unilateral and contralateral unaffected were found age matched (pairs 5–10, see below). The mean foot length for a patient was calculated using the average of toe lengths T1–T5. The comparison of foot size in ≤ 36 and >36 months bracing durations was made only in patients with unilateral clubfoot (pairs 11–14). The following pairs of measurements were compared:

- Pair 1: Mean foot length between bilateral affected feet (*n* = 49 each).
- Pair 2: Foot width between bilateral affected feet (*n* = 49 each).
- Pair 3: Mean foot length between unilateral affected and the contralateral unaffected feet (*n* = 51 each).
- Pair 4: Foot width between unilateral affected and the contralateral unaffected feet (*n* = 51 each)
- Pair 5: Mean foot length between bilateral (counted as two) (*n* = 98) and unilateral affected (*n* = 51) feet. These were found age matched.
- Pair 6: Foot width between bilateral (counted as two) (*n* = 98) and unilateral affected (*n* = 51) feet. These were found age matched.
- Pair 7: Mean foot length of bilateral (counted as two) and unilateral affected (*n* = 149) versus unaffected (*n* = 51) feet. These were found age matched.
- Pair 8: Foot width of bilateral (counted as two) and unilateral affected (*n* = 149) versus unaffected (*n* = 51) feet. These were found age matched.
- Pair 9: Mean foot length between bilateral (counted as two) (*n* = 98) and unaffected (*n* = 51) feet. These were found age matched.
- Pair 10: Foot width between bilateral (counted as two) (*n* = 98) and unaffected (*n* = 51) feet. These were found age matched.
- Pair 11: Mean foot length of children using brace with unilateral affected versus contralateral unaffected feet with follow up of upto 36 months post tenotomy (*n* = 38 each).
- Pair 12: Foot width of children using brace with unilateral affected versus contralateral unaffected feet with follow up of upto 36 months post tenotomy (*n* = 38 each).
- Pair 13: Mean foot length of children completing brace protocol with unilateral affected versus unaffected feet with follow up of beyond 36 months post tenotomy (*n* = 13 each).
- Pair 14: Foot width of children completing brace protocol with unilateral affected versus unaffected feet with follow up of beyond 36 months post tenotomy (*n* = 13 each).

The data were recorded on Microsoft Excel^®^ and statistical relationships between measurements were calculated using paired *t* tests on online GraphPad software^®^.

## Results and interpretations

The age range in 100 patients was 5–132 months (average 32.5 months). The male female proportion was approximately 3:1. Average follow up was 22.2 months (range, 3–88 months). In the unilateral group, out of 51 patients, 13 had more than 3 years of follow up. The measurements and statistical significance are recorded as Table 1.

**Table 1:** Comparison of various pairs and their statistical significance*.

Pairs	Measurements in cm		*P* value
Pair 1	Right:12.2 ± 2.2	Left:12.1 ± 2.2	0.15
Pair 2	Right:5.1 ± 0.7	Left:5.2 ± 0.8	0.05
Pair 3	Affected:12.5 ± 2.2	Unaffected:13.0 ± 2.4	0.27
Pair 4	Affected:5.3 ± 0.7	Unaffected:5.4 ± 0.7	0.53
Pair 5	Bilateral:12.2 ± 2.2	Unilateral:12.5 ± 2.2	0.42
Pair 6	Bilateral:5.2 ± 0.7	Unilateral:5.3 ± 0.7	0.15
Pair 7	Total affected:12.3 ± 2.2	Unaffected:13.0 ± 2.4	***0.03***
Pair 8	Total affected:5.2 ± 0.7	Unaffected:5.4 ± 0.7	***0.02***
Pair 9	Bilateral:12.2 ± 2.2	Unaffected:13.0 ± 2.4	***0.03***
Pair 10	Bilateral:5.2 ± 0.7	Unaffected:5.4 ± 0.7	***0.03***
Pair 11	Unilateral upto 36 months:11.7 ± 1.7	Unaffected upto 36 months:12.1 ± 1.8	0.21
Pair 12	Unilateral upto 36 months:5.1 ± 0.6	Unaffected upto 36 months:5.2 ± 0.7	0.66
Pair 13	Unilateral beyond 36 months:15.0 ± 1.7	Unaffected beyond 36 months:15.7 ± 1.9	0.37
Pair 14	Unilateral beyond 36 months:5.9 ± 0.6	Unaffected beyond 36 months:6.1 ± 0.6	0.5

* paired *t* test

As depicted in Table 1, we found bilateral feet were similar in size (pairs 1 and 2). The unilateral affected feet matched in size with unaffected feet (pairs 3 and 4). Even the size difference between bilateral and unilateral affected feet was not significant (pairs 5 and 6). When we compared overall clubfoot size versus unaffected feet after age matching, a statistical significant difference was apparent (in length; pair 7, *p* = 0.03 and in width; pair 8, *p* = 0.02). On the whole, the clubfeet on an average were 0.7 cm (5.3%) shorter than the unaffected feet. The bilateral feet were significantly smaller than age matched unilateral unaffected feet [in length 0.8 cm (6.1%); pair 9, *p* = 0.03 and in width 0.2 cm, (3.7%); pair 10, *p* = 0.03].

The Ponseti managed unilateral foot size was comparable with unaffected foot during the bracing duration (pair 11 and 12) and the size comparability was maintained even after bracing protocol was over i.e. beyond 36 months (pair 13 and 14).

## Discussion

Our study provided the quantitative evidence that Ponseti method has fulfilled the promise of a near equal sized feet post correction. Previous studies have compared results of conservative and surgically corrected clubfeet but none quantified the foot sizes of children treated exclusively by Ponseti method of serial casting [8,9,11]. Kesemenli et al. published the results of anthropometric measurements in unilateral clubfeet [8]. They divided their patients into three groups: conservatively treated; surgically treated; and conservatively treated on one side and surgically treated on the other. The average age in author's series was 8.8 years (range, 7–12 years). The conservatively treated foot with follow up of mean 9 years was on average of 0.91 cm (0.4–2.1 cm) shorter in length than the unaffected foot (*P* < 0.01). The foot width again was shorter by average 0.05 cm (0.1–0.4 cm) (*P *> 0.05). But the conservative non-operative casting method utilized by them was not Ponseti [8]! Another study (2007) investigating bilateral clubfoot cases (*n* = 92), concluded that these feet tend to be equal in length, with a mean difference in length of only 3.76% (SD 2.38) [11]. However, the study was too heterogeneous in methods of clubfoot treatment, and moreover, the Ponseti method was not used in any of the treatment regimen [11].

Recently, Gamble et al. published a longitudinal study comparing children with unilateral clubfeet treated by posterior medial release (*n* = 65) and Ponseti method (*n* = 28) [9]. The average follow up was 68 months. Along with the above groups which revealed no statistical difference in foot size [8.70% (95%CI 7.54–9.87%)], the authors also described the comparison between the length of the feet managed by Ponseti method and the unaffected feet. Children with a unilateral clubfoot had 10% smaller foot length as compared to the unaffected side. The authors concluded the smaller foot size is intrinsic to the condition and not due to type of treatment.

In our series, unilateral foot matched the unaffected and the bilateral with their opposites, anthropometrically. Gray et al. have observed that patients with bilateral clubfeet have similar baseline deformity, response to initial Ponseti treatment, Achilles tenotomy rate, and relapse outcomes [12]. The authors cautioned against use of bilateral presentations as independent data points in research calculations [12]. Her team's other observation that bilateral feet are potentially more severe than unilateral clubfeet with odds ratio 2.6 (95% confidence interval 1.3–5.1) was also reciprocated in our study [13]. Age matched bilateral feet were significantly shorter in size compared to unaffected feet in our series too. This indirectly supports the finding that an initial smaller foot persists to some extent post treatment also. However, the measured differences in the intergroup bilateral or with unaffected side were not more than 0.8 cm and did not warrant an altered shoe size for our patients.

We also investigated whether the Ponseti managed unilateral foot size was comparable with unaffected foot during the bracing duration (pair 11 and 12) and was the comparability maintained even after bracing protocol was over i.e. beyond 36 months (pair 13 and 14). This was important as most Ponseti centres practice a brace protocol of 3–4 years post correction. The affirmative findings in our study leads to indirect inference that the affected foot gets comparable dimensions to unaffected foot immediately following Ponseti correction and the same is maintained during bracing and post bracing period. The longest follow up for unilateral patients in our series had been 88 months.

There were several limitations to our study which we acknowledge. There were chances of interobserver variations in foot size measurements which were minimized by utilizing a single operator and same measurement tool for all the patients. The patients were still young and foot size changes can be dictated by child's growth, culture, ethnicity, sports activities and exercise regime. The brace compliance and duration of SFAB wear were assumed to be nonconfirming for the series.

The strength of our study is that it is a probably first of its kind, large, dedicated anthropometric study of corrected clubfeet in Ponseti era. It shows the near normalcy of the treated foot (unilateral versus unaffected foot and inter-bilateral) on quantitative basis rather than assumptions. The scientific study will help dispense the myths being circulated on several internet sites. Our pilot study also suggests that the intrinsic factors present in a clubfoot rather than the treatment with Ponseti method are probably responsible for foot size differences observed.

## Conclusions

Post Ponseti treatment, idiopathic bilateral feet were similar in size and, the unilateral affected feet matched in dimensions with unaffected feet. Even the size difference between bilateral and unilateral affected feet was not significant. The overall clubfeet size especially those with bilateral disease, was significantly shorter than unaffected side. The Ponseti managed unilateral foot was comparable with unaffected foot size during the bracing duration and this comparability was maintained even after bracing protocol was over i.e. beyond 36 months.

## Conflict of interest

AA and AR certify that they have no financial conflict of interest (e.g., consultancies, stock ownership, equity interest, patent/licensing arrangements, etc.) in connection with this article.
